# Incidence of sepsis following transrectal ultrasound guided prostate biopsy at a tertiary-care medical center in Lebanon

**DOI:** 10.1590/S1677-5538.IBJU.2014.0607

**Published:** 2016

**Authors:** Mohammed Shahait, Jad Degheili, Fadi El-Merhi, Hani Tamim, Rami Nasr

**Affiliations:** 1 Department of Surgery, American University of Beirut Medical Center, Beirut, Lebanon;; 2 Department of Radiology, American University of Beirut Medical Center, Beirut, Lebanon;; 3 Department of Internal Medicine and Clinical Research Institute, American University of Beirut Medical Center, Beirut, Lebanon

**Keywords:** Prostate, Biopsy, Ultrasonography, Sepsis, Neoplasms

## Abstract

**Background:**

Urosepsis is a rare but life-threatening complication following transrectal ultrasound (TRUS) guided needle prostate biopsy. Despite the technological and pharmacological improvements, the problem of bacterial urosepsis after prostate biopsy remains. A strategy for preventing urosepsis following TRUS prostate biopsy in areas with high prevalence of resistant strains or patients presenting risk factors is lacking.

**Objectives:**

The aim of this study was to assess the prevalence of urosepsis, as well its predictors, following TRUS guided needle biopsy of the prostate in a tertiary care medical center in Lebanon.

**Materials and Methods:**

We carried out a retrospective study on all patients who underwent TRUS prostate biopsy at the American University of Beirut Medical Center between January 1, 2011 and June 31, 2013. Patients’ hospital charts were reviewed. Data collected included demographic information, pre-procedure disease specific information, as well as post-procedure information. Predictors of urosepsis following TRUS were assessed.

**Results:**

In total, 265 patients were included in this study, where the prevalence of urosepsis following TRUS prostate biopsy was found to be 9.4%. The significant independent predictors of urosepsis were found to be: age with an OR=0.93 (95% CI: 0.88–1.00, p-value=0.03), and hypertension comorbidity with an OR=3.25 (95% CI: 1.19–8.85, p-value=0.02).

**Conclusion:**

We found a high prevalence of urosepsis among patients who have undergone TRUS prostate biopsy, and identified two significant risk factors. The results of this study highlight the importance of implementing strategies for prevention of urosepsis following TRUS prostate biopsy.

## INTRODUCTION

Prostate cancer is the second most commonly diagnosed cancer in men and represents a significant health problem (1). A total of 233,000 new cases of prostate cancer and 29,480 deaths from the disease are anticipated in the United States in 2014 (2). The highest incidence rates f prostate cancer are reported in Australia/New Zealand, Western and Northern Europe and North America, largely because of the availability of screening programs and the widespread use of prostate-specific antigen (PSA) testing in those regions (3). In Lebanon, the incidence rates for prostate cancer increased during the period 2003–2008 from 29.9 to 39.2 cases per 100,000, and became the most-reported cancer in 2008 (4).

Transrectal ultrasound (TRUS)–guided prostate biopsy remains the gold standard technique to confirm the diagnosis of prostate cancer (5). According to recent estimates, approximately more than 1 million TRUS biopsies are performed per year in Europe and the United States (6). Although TRUS biopsy is generally considered to be a relatively low-risk outpatient procedure, post-biopsy complications and hospital admissions have increased at alarming rates during the last decade due to an increasing rate of infection related complications (7). TRUS biopsy complication rates are reported in up to 50% of cases and range from minor complications, such as hematuria, hematospermia or rectal bleeding, acute urine retention to much more severe complications, such as anemia, fainting, febrile urinary infections, syncope, and even septic shock. The infectious complications, which range from bacteriuria to sepsis, affect 1-4% of the patients who undergo this procedure (8). One study from Ontario, Canada reported that the hospital admission rate for infection-related complications within 30 days of the procedure increased from 1.0% in 1996 to 4.1% in 2005 (9). The reported incidence of urinary tract infections (UTI) after TRUS biopsy typically ranges between 2% and 6% with approximately 30%-50% of these patients having accompanying bacteremia (10). Severe sepsis has been described in 0.1%-3.5% of cases after TRUS biopsy (9). The proposed mechanism of infection is likely the introduction of bacteria into the bladder and bloodstream from the rectum (11). The most common organism responsible for these infectious complications is E. coli (7, 12). Moreover, the spread of multiresistant E. coli is of particular concern (13). Resistant bacteria are more prevalent in some countries. Antibiotic overuse or misuse has been blamed, but a wider dissemination of resistant organisms resulting from globalization and international travel may also be a factor (13, 14).

Factors that may predict which men are at greatest risk of infectious complications are: underlying medical comorbidities, particularly diabetes mellitus, presence of urethral catheter, and recent hospitalization (15, 16).

Infectious complications can be serious, requiring effective preventative strategies and prompt management. Different methods have been studied for reducing the rate of infection following TRUS guided biopsy such as the use of prophylactic antibiotic, bowel cleansing enema, and using disposable instruments. Antibiotic prophylaxis is the only measure that has been shown to reduce the rate of infection post TRUS in randomized controlled trial setting, (17, 18). The American Urological Association (AUA) and the European Association of Urology guidelines for antibacterial prophylaxis for TRUS prostate biopsies recommend fluoroquinolones as agents of first choice due to their broad spectrum of activity, excellent penetration into prostatic tissue, and their prolonged post-antibiotic effect (19). The AUA guidelines also recommend aminoglycosides or aztreonam with metronidazole or clindamycin as alternatives to fluoroquinolones (17).

Fluoroquinolone-resistant E.coli is emerging globally (20). This poses a clinical challenge to the urologists to tailor the prophylactic regimen according to the resistance pattern in their hospitals. Multiple studies pointed toward the feasibility of using rectal swab culture to guide the prophylactic antibiotic regimen (21). In a survey of 3355 urologists in United States of America, Joel et al. reported 14 different duration of treatment using 10 different classes of antibiotic (22).

The aim of this study was to assess the prevalence of urosepsis following transrectal ultrasound guided needle biopsy of the prostate, as well as its predictors in a tertiary-care medical center in Lebanon.

## MATERIALS AND METHODS

### Study design and setting

We carried out a retrospective chart review on all patients who underwent TRUS prostate biopsy at the American University of Beirut Medical Center between January 1, 2011 and June 31, 2013.

### Inclusion/exclusion criteria

Patients eligible to be included in the study were those undergoing TRUS prostate biopsy, and who had no clinical evidence of prostatitis and had a negative urine culture prior to the biopsy.

### Ethical considerations

The institutional review board at the American University of Beirut Medical Center approved the study.

### Procedure

During the study period, TRUS prostate biopsy was performed by four urologists in the Radiology Department at the American University of Beirut Medical Center. All patients received prophylactic antibiotics. The adopted regimen by all urologists consisted of a flouroquinolone orally to be started one day prior to the procedure and gentamicin IV or IM injection 30 minutes before the procedure. The procedure was performed while the patient was in the left lateral decubitus position. The anus and perineum were wiped with iodine swabs. A 5 to 9 MHz probe covered by a sterile condom and sterile K-Y Gel was introduced into the rectum and used to measure the prostate size and guide the local anesthesia injection and needle biopsies. An 18 French disposable gun and needle were used, in comparison to previous years, where we used a re-sterilizable automatic gun with a disposable needle. A standard sextant set of biopsies were taken, with the addition of targeted cores as needed to any suspicious lesion.

### Outcome

The endpoint in our study was the development of urosepsis after TRUS prostate biopsy. Urosepsis was defined as urinary symptoms, leukocytosis, and/or fever more than 38.0°C orally.

### Data collection

Other information collected in this study included demographic information (such as age), lifestyle information (such as smoking), comorbidities (such as cardiac, hypertension, and diabetes), and pre-procedure disease specific information (including prostate size, post-void residual (PVR), PSA value and ratio, presenting urinary symptoms, recent treatment with antibiotic, positive urine analysis, bowel preparation, and previous biopsies). Moreover, post-procedure information were also collected, and included: positive urine analyses, urine culture and the bacteriology, as well as days to develop urosepsis.

## Statistical analyses

Data entry and statistical analyses were performed using the Statistical Package for Social Sciences (SPSS), version 21.0. Descriptive analyses were carried out by reporting the number and percent for categorical variables, whereas the mean and standard deviation were calculated for continuous ones. Associations between the different risk factors and the development of urinary tract infection were assessed using the chi-square test for categorical variables or the student’s t-test for continuous ones.

To account for the potential confounding effect of the different risk factors on the development of the outcome, multivariate logistic regression analyses were carried out. Included in the model were the multiple variables that could affect urinary infection post biopsy. These variables included: age, smoking status, co-morbidities (such as hypertension and diabetes), prostate size, previous urine analysis, antibiotics use, and mechanical bowel prep.

## RESULTS

The demographic characteristics, as well as the pre-and post-procedure information for the total sample (n=265) are presented in Table-1. The average age of the patients was 64.4 years (sd=7.9), where almost half of them were nonsmokers (53.9%). The prevalence of cardiac diseases, hypertension, and diabetes were 13.6%, 38.6%, and 15.2%, respectively. The prostate size prior to the procedure was found to be 49.9 (sd=29.2) and the PSA ratio was 0.2 (sd=0.1). Overall, 58 patients received antibiotics before the prophylactic dose for their lower urinary tract symptoms or as empirical treatment of high PSA (21.9%) and 26.8% had bowel preparation. As for the outcomes, the incidence of urosepsis was 9.4% (25 patients). The urine culture was positive for 18 patients (6.8%), where 13 had E. coli resistance (72.2%). Three patients had E.coli sensitive (16.7%) and 2 patients had Klebsiella pneumoniae (11.1%). The average number of days to develop sepsis was 7.6 days (sd=18.2).

**Table 1:** Baseline characteristics of the population.

	Variables		n (%)
Total sample			N=265
Demographic	Age	Mean (±sd)	64.4 (±7.9)

Lifestyle	Smoking	Non smoker	117 (53.9%)
		Smoker	52 (24.0%)
		Ex-smoker	48 (22.1%)

Comorbidities	Cardiac	No	228 (86.4%)
		Yes	36 (13.6%)
	HTN	No	162 (61.4%)
		Yes	102 (38.6%)
	Diabetes	No	224 (84.8%)
		Yes	40 (15.2%)

Pre-procedure	Prostate size	Mean (±sd)	49.9 (±29.2)
	PVR	Mean (±sd)	53.5 (±83.5)
	PSA number	Mean (±sd)	50.0 (±379.7)
	PSA ratio	Mean (±sd)	0.2 (±0.1)
	Symptoms	No	107 (40.4%)
		Yes	158 (59.6%)
	Antibiotics	No	207 (78.1%)
		Yes	58 (21.9%)
	Urine analysis(positive)	No	252 (95.1%)
		Yes	13 (4.9%)
	Enema	No	194 (73.2%)
		Yes	71 (26.8%)
	Biopsy	No	246 (92.8%)
		Yes	19 (7.2%)

Post-procedure	Urine analysis(positive)	No	243 (91.7%)
		Yes	22 (8.3%)
	Sepsis	No	240 (90.6%)
		Yes	25 (9.4%)
	Urine culture	No	247 (93.2%)
		Yes	18 (6.8%)
	Urine culture (bacteriology)	E.choli sensitive	3 (16.7%)
		E.choli resistant	13 (72.2%)
		Klebsiella Pneumoniae	2 (11.1%)
	Hospital admission	No	245 (96.1%)
		Yes	10 (3.9%)
	Days to develop sepsis	Mean (±sd)	7.6 (±18.2)

Table-2 summarizes the association between the different patient characteristics and the development of urosepsis. Patients who developed urosepsis were younger (mean=62.8 years, sd=6.3 versus non-septic patients whose age was 64.5, sd=8.0), and more likely to be smokers (33.3% versus non-septic patients, 2.8%), although the association was not significant for both characteristics, p-value=0.31 and 0.51, respectively. Patients who developed urosepsis were more likely to be hypertensive (64.0%) compared to the non-septic ones (36%), with a p-value of 0.006. Similarly, uroseptic patients were more likely to be diabetic (32.0%) as compared to non-septic patients (13.4%), p-value=0.03. As for the pre-procedure information, none of the assessed characteristics was found to be significantly different between the uroseptic and non-septic patients. For instance, uroseptic patients were less likely to have had bowel preparation (16.0%) as compared to the non-septic ones (27.9%), p-value=0.2.

**Table 2:** Association between different factors and urosepsis

	Variables		All n (%)	No sepsis n (%)	Sepsis n (%)	P value
Total sample			N=265	N=240	N=25	
Demographic	Age	Mean (±sd)	64.4 (±7.9)	64.5 (8.0)	62.8 (6.3)	0.31

Lifestyle	Smoking	Non smoker	117 (53.9%)	106 (54.9%)	11 (45.8%)	0.51
		Smoker	52 (24.0%)	44 (2.8%)	8 (33.3%)	
		Ex-smoker	48 (22.1%)	43 (22.3%)	5 (20.8%)	

Comorbidities	Cardiac	No	228 (86.4%)	207 (86.6%)	21 (84.0%)	0.76
		Yes	36 (13.6%)	32 (13.4%)	4 (16.0%)	
	HTN	No	162 (61.4%)	153 (64.0%)	9 (36.0%)	0.006
		Yes	102 (38.6%)	86 (36.0%)	16 (64.0%)	
	Diabetes	No	224 (84.8%)	207 (86.6%)	17 (68.0%)	0.03
		Yes	40 (15.2%)	32 (13.4%)	8 (32.0%)	

Pre-procedure	Prostate size	Mean (±sd)	49.9 (±29.2)	49.3 (±27.9)	54.8 (±39.8)	0.39
	PVR	Mean (±sd)	53.5 (±83.5)	54.8 (±88.4)	45.0 (±36.2)	0.60
	PSA number	Mean (±sd)	50.0 (±379.7)	50.7 (±396.0)	44.4 (±179.2)	0.94
	PSA ratio	Mean (±sd)	0.2 (±0.1)	0.2 (±0.1)	0.2 (±0.1)	0.14
	Symptoms	No	107 (40.4%)	101 (42.1%)	6 (24.0%)	0.08
		Yes	158 (59.6%)	139 (57.9%)	19 (76.0%)	
	Antibiotics	No	207 (78.1%)	188 (78.3%)	19 (76.0%)	0.79
		Yes	58 (21.9%)	52 (21.7%)	6 (24.0%)	
	Urine analysis	No	252 (95.1%)	228 (95.0%)	24 (96.0%)	1.00
		Yes	13 (4.9%)	12 (5.0%)	1 (4.0%)	
	Enema	No	194 (73.2%)	173 (72.15)	21 (84.0%)	0.20
		Yes	71 (26.8%)	67 (27.9%)	4 (16.0%)	
	Biopsy (previous)	No	246 (92.8%)	222 (92.5%)	24 (96.0%)	1.00
		Yes	19 (7.2%)	18 (7.5%)	1 (4.0%)	

Finally, Table-3 summarizes the results of the multivariate analyses carried out to identify the predictors of urosepsis among patients who underwent TRUS prostate biopsy. Age was found to be a significant predictor, where older patients are less likely to have urosepsis (OR=0.93, 95% CI: 0.88-1.00, p-value=0.03). Hypertension status was also found to be significantly associated with urosepsis (OR=3.25, 95% CI: 1.19-8.85, p-value=0.02). Smokers, diabetics, and patients with symptomatic presentation were at higher chance of developing urosepsis. On the other hand, patients who had bowel preparation or cardiac disease were less likely to develop urosepsis; but none of these associations was statistically significant.

**Table 3:** Multivariate analysis for the predictors of urosepsis (Cox regression model).

Predictors		Adjusted OR (95%CI)	P value
Age		0.93 (0.88 – 1.00)	0.03

Smoking	Non smoker	Reference	Reference
	Smoker	1.56 (0.55-4.45)	0.40
	Ex-smoker	0.95 (0.30-3.07)	0.93

Cardiac		0.92 (0.27-3.19)	0.90
HTN		3.25 (1.19-8.85)	0.02
Diabetes		2.18 (0.79-6.01)	0.13
Prostate size		1.00 (0.99-1.02)	0.71
Symptoms		1.83 (0.66-5.04)	0.24
Enema		0.55 (0.17-1.79)	0.32

All patients who developed urosepsis were admitted to the hospital for intravenous antibiotic and monitoring. All patient received carbapenem antibiotic for 14 days at least. The average number of days of hospitalization was 5.

## DISCUSSION

TRUS prostate biopsy is the standard test to diagnose prostate cancer after a suspicious digital rectal exam and elevated PSA. Occasionally this procedure is associated with significant morbidity. In this retrospective study, charts of patients who underwent TRUS prostate biopsy at the American University of Beirut Medical Center between January 1, 2011 and June 31, 2013 were reviewed to assess the prevalence of urosepsis following transrectal ultrasound (TRUS) prostate biopsy, as well as its predictors. We found that the prevalence of (TRUS) prostate biopsy urosepsis to be 9.4%. Multivariate analysis identified age and hypertension comorbidity to be significantly associated with an increased risk of developing urosepsis following TRUS prostate biopsy.

The prevalence of urosepsis following TRUS prostate biopsy found in our study (9.4%) was higher than that reported in other studies. The frequency of urosepsis varied among those studies between 0.2% and 3.06%. The lowest rates of urosepsis were reported by Zaytoun et al. in a North American cohort, and Raaijmakers et al. in a European Randomized Study, who reported urosepsis prevalence rates of 0.2%, and 0.5%, respectively (17, 23). However, other series of studies carried out by Carmignani et al., Akduman et al., and Simsir et al. in reported higher rates of urosepsis (2.2%, 3.0%, and 3.06%, respectively) (16, 24, 25). Only one Asian study conducted by Raheem et al. in 2012 reported no septic complications (26). This variation in rates of sepsis among different studies arises from differences in biopsy techniques, prophylactic protocols, consistent reporting, and the definition of urosepsis used. The prophylactic regimes preventing infectious complications may differ with respect to the use of an antibiotic (type of antibiotic used, dose, and method of administration and duration of the therapy), as well as whether a cleansing rectal enema was used or not (24). Other factors that may increase the antibiotic resistance and thus increasing the rate of sepsis by an antibiotic resistant strain are past history of hospitalization, a past history of exposure to antibiotics, a past history of catheterization, and a past history of urogenital surgery (27, 28). The proponents of the high sepsis rate in our study are cited as following: the high prevalence of E. coli resistant to fluoroquinolone in Lebanon which was noted in a recent review of the patterns and trends of bacterial resistance to antimicrobial agents over the last decade in our center, self-medication with antibiotics which is a frequent problem in Beirut area, polypharmacy in patients with co-morbidities which may affect the antibiotic efficacy (29, 30).

As for the risk factors significantly associated with urosepsis following TRUS prostate biopsy, age was found to be a significant risk factor OR=0.93 (95% CI: 0.88–1.00, p-value=0.03), where older patients were less likely to have urosepsis. One possible reason may be that younger patients are more likely to self-report complications compared to older patients. Few studies in the literature reported on the effect of age on sepsis after TRUS prostate biopsy. In a study carried out by Lee et al. between 2003 and 2006 reported no significant difference in the urosepsis rate in relation to age (p-value=0.82) after TRUS prostate biopsy (29). However, several studies reported on higher incidence of general complications after TRUS biopsy in younger patients (30).

Another factor found to be significantly associated with urosepsis following TRUS prostate biopsy in our study was hypertension comorbidity with an OR=3.25 (95% CI: 1.19-8.85, p-value=0.02). The study carried out by Lee et al. did not report any significant association between hypertension and sepsis following TRUS prostate biopsy (p-value=0.18) (29). Further comparison of this association with the literature is hard due to the limited availability of studies assessing this association.

Although many urologists intuitively assume that increased post void residue would predispose to development of post TRUS infection, clear evidence is lacking as well. Our data did not show any significant difference in the rate of urosepsis among patients with post void residue verses those with no significant residue.

Finally, we should emphasize the limitations of this study, including the small number of patients. In addition, the clinical results were analyzed retrospectively based on charts review.

Notwithstanding those limitations, this study elucidates of the impact of increasing bacteria resistance prevalence on the rate of post TRUS urosepsis.

## CONCLUSIONS

Urosepsis after TRUS biopsy represents a great challenge for urologists; sometimes its risks are more important than its benefits. It tips the balance between the risk and the benefit of prostate screening. Implementation of new strategies to prevent urosepsis and early treatment is required; especially in areas where bacterial resistance is endemic. We are considering revisiting our prophylactic regimen in our institution, and developing a follow-up system in which the patients will be contacted every 48 hours by a physician assistant to be screened for early urosepsis symptoms. Larger studies that explore the risks of urosepsis after TRUS biopsy are required. Moreover, identifying biomarkers that are associated with developing urosepsis after TRUS biopsy represents a unique research opportunity.
